# Impact of the 2023 armed conflict on Sudan's healthcare system

**DOI:** 10.1002/puh2.134

**Published:** 2023-10-28

**Authors:** Alhadi Khogali, Anmar Homeida

**Affiliations:** ^1^ Health Management Support Team Amsterdam the Netherlands; ^2^ Faculty of Medicine University of Gezira Wad Medani Sudan

**Keywords:** armed conflict, health system, Sudan

## Abstract

The armed conflict in Sudan erupted in mid‐April 2023, resulting in many casualties, internal displacement, and refugees. Sudan's health system was severely affected by multiple attacks on healthcare facilities and workers, closure of hospitals, and occupation of medical facilities by armed groups. Critical services such as immunization, nutrition, and emergency care have been suspended, disrupting health services in conflict states and increasing strain on neighboring states' healthcare facilities. The conflict also caused shortages of essential medical supplies, looting of healthcare facilities and humanitarian supplies, and destruction of infrastructure, affecting the supply chain and availability of healthcare resources. The deployment and distribution of the health workforce have become challenging and may lead to a further brain drain of healthcare professionals. The financial challenges in healthcare financing are expected to worsen due to the conflict's economic impact on fiscal space and fiscal capacity. The health system's governance has been affected by a leadership vacuum, and decision‐making may shift to the state level. The conflict has also exacerbated the burden of diseases, including communicable diseases, malnutrition, and mental health issues, with potential outbreaks of dengue fever, measles, and spikes of gender‐based violence reported. In response to the conflict, the restoration and maintenance the health system is pivotal via coordinating efforts between the Sudanese Ministry of Health and international partners. The safety of healthcare workers and the delivery of essential supplies need to be restored via strengthening compliance with international humanitarian law, pooling of funds, and services at primary care level. This commentary discusses the impact of the 2023 conflict on Sudan's health and health system, particularly on different building blocks of Sudan's health system as well as the burden of disease and humanitarian response priorities.

## INTRODUCTION

**FIGURE 1:**
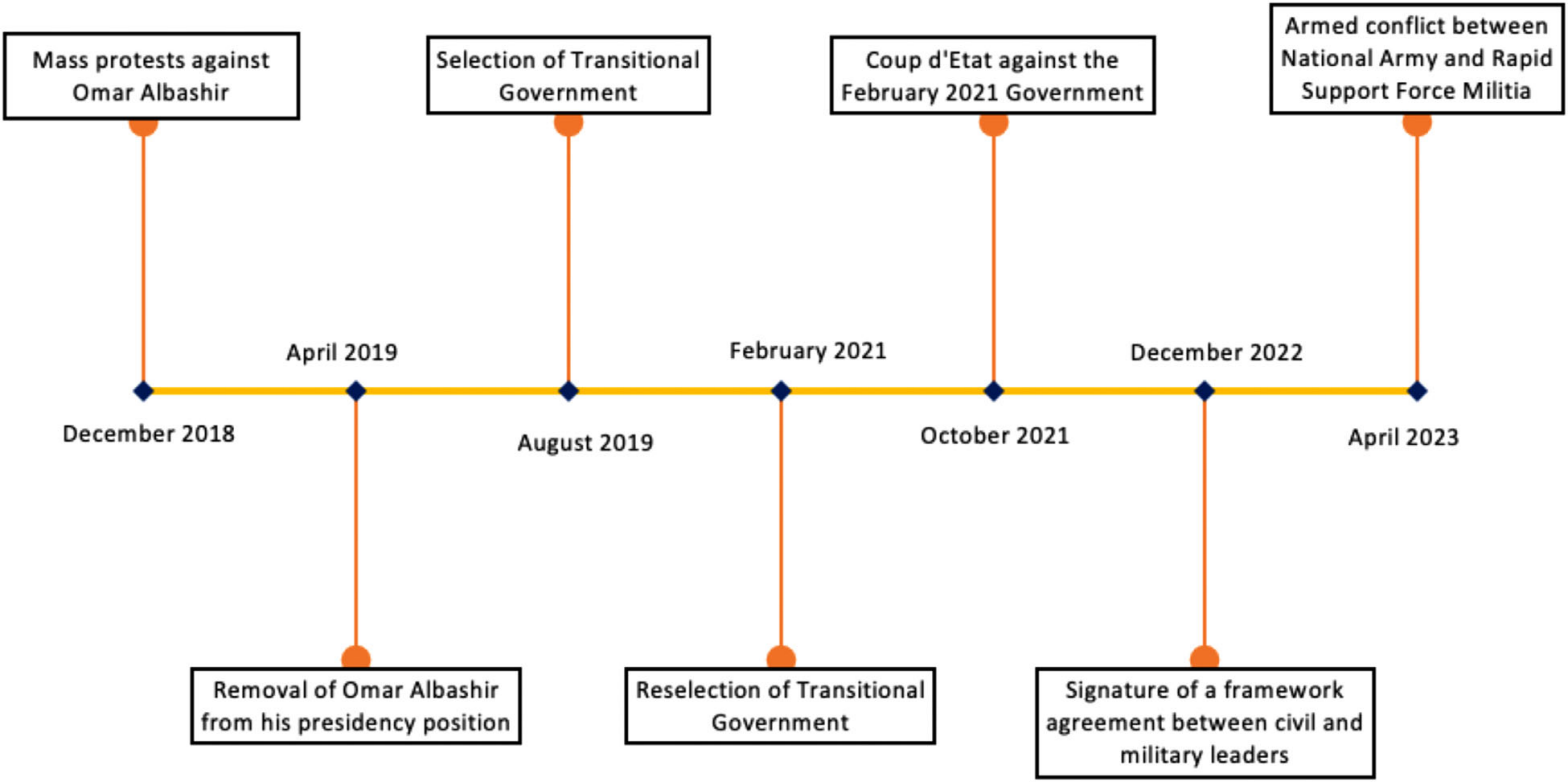
Key political milestones in the recent history of Sudan, December 2018–April 2023.

Armed conflicts lead to the collapse of health systems. It has negative effects on human resources for health and overloads hospital capacities. As of August 2023, there are active armed conflicts in 20 countries in the world, mostly in Asia and Africa. Sudan has been in a state of chronic political unrest since December 2018 (Figure 1), and an armed conflict erupted on April 15, 2023, between the National Army and the Rapid Support Force (RSF) Militia, resulting in over 1133 deaths, 11,796 injuries, 2.2 million cases of internal displacement, and 615,000 refugees to other countries as of July 2023 [1].

**FIGURE 2:**
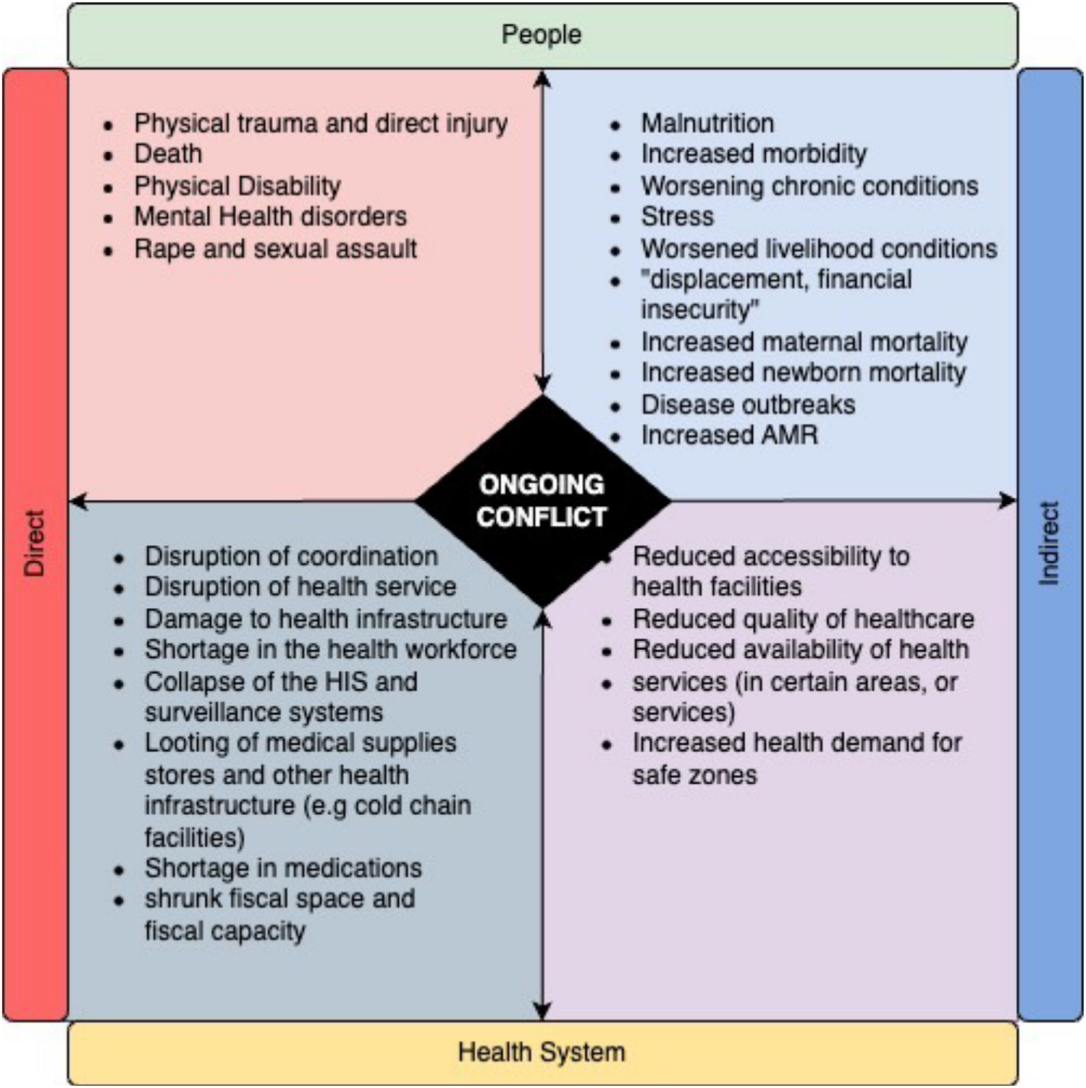
Overview of the direct and indirect impacts of the conflict on the health system and the people's health in Sudan.

Preserving the health system in Sudan in the early stages of the war is crucial, as estimates suggest that during conflicts indirect causes of mortality is considerable compared to direct causes mortality with a ratio of 11:4 [2]. This commentary aims to describe the impact of the 2023 armed conflict on Sudan's health system, with a focus on several WHO building blocks and the burden of disease while proposing a key consideration for the response to the current situation in Sudan (Figure 2).

## HEALTH SERVICE DELIVERY

Several hospitals and health facilities in Khartoum were closed as a result of the occupation of hospitals by RSF, systematic looting, and shortages in supply and resources. The Sudanese Federal Ministry of Health (FMoH) has reported multiple attacks on healthcare and humanitarian workers, leading to the closure of hospitals, especially in Khartoum—WHO verified 46 of these attacks [3]. RSF has also evicted medical staff and taken control of several hospitals in Khartoum using them as military outposts. Critical services were suspended, including immunization, nutrition services, baby deliveries, emergency surgical operations, intensive care, and renal dialysis [4]. Patients’ loads were diverted to health facilities in other states following the massive displacement movements. Yet, there was no concurrent expansion of services to accommodate those newly displaced. The disruption of health services and the burden on health facilities in nearby states is expected to continue if this civil war lasts longer.

## MEDICINES AND SUPPLY CHAIN

At the beginning of the conflict, there were reports from hospitals in Khartoum receiving injured people on shortages of blood, fluids, and other life‐saving commodities along with shortages of water, electricity, fuel, and food for patients [5]. This was concurrent with the directed destruction of more than 1 million polio vaccines; cold chain facilities and immunization programs premises. Aid organizations have documented incidences of looting; for example, World Food Program reported the loss of 13–14 million USD value of supplies [6]. Some countries have responded by sending emergency medical supplies that have arrived in Port Sudan, but there is no evidence that these supplies were delivered to health facilities in other states.

## HUMAN RESOURCES FOR HEALTH (HRH)

All universities and other academic institutes in Sudan were forced to shut down, leading (and will lead) to further delays in the production of the different healthcare cadres. Moreover, the deployment and distribution of the health workforce became difficult; the aforementioned occupation of hospitals by RSF has ultimately led medical doctors and other health professionals to leave hospitals. Doctors and other professionals who remained had to work under very difficult and stressful conditions, supply shortages, and lack of safety—threatening their own lives to save patients. Arguably, this conflict will increase the brain drain of Sudanese healthcare professionals, who may seek better financial and professional opportunities, and careers abroad (viz., in the Gulf, Europe, the Americas, and Australia) [7].

## HEALTH FINANCE

Prior to this ongoing conflict, the financing healthcare services were challenging—with rising out‐of‐pocket expenditures and falling government financial contributions. In addition, the country was already undergoing sweeping financial reforms which resulted in economic stresses and rising inflation [7]. This conflict is likely going to the shrink fiscal space and fiscal capacity caused by the massive damage to the infrastructure and probably the increased investment in weaponry and military priorities at the expense of health and other development priorities. Nonetheless, in response to the health priorities, the humanitarian partners are actively working in Sudan to secure additional resources to be able to scale up their responses across the country [8].

## GOVERNANCE

Sudan enters this conflict with a longstanding health leadership vacuum [7]. Prior to this conflict, the FMoH undersecretary served as an acting minister, whereas the TSC served as the parliament. In the current absence of clear nationally driven policy direction, the health system is (and will likely be) operating in an auto‐pilot mode. Gradually, there will be minimal input on a federal level, and decision‐making may take place at the state level. To mitigate this vacuum, FMoH is actively engaging with health partners on the ground to ensure availing the basic services and help in guiding the health partners' interventions to be of an asset for planned interventions through two operation hubs in Port Sudan and Wad Medani cities as a central coordination platform.

The role of civil society in supporting the health system has grown over the last decade, especially in the surveillance and response to several local outbreaks and the COVID‐19 pandemic. However, the capacity of civil society to respond to the ongoing conflict remains unknown. Currently, less than 20% of the Sudan Humanitarian Fund has been allocated to support the local actors and community groups on the ground [1].

## BURDEN OF DISEASES

**FIGURE 3:**
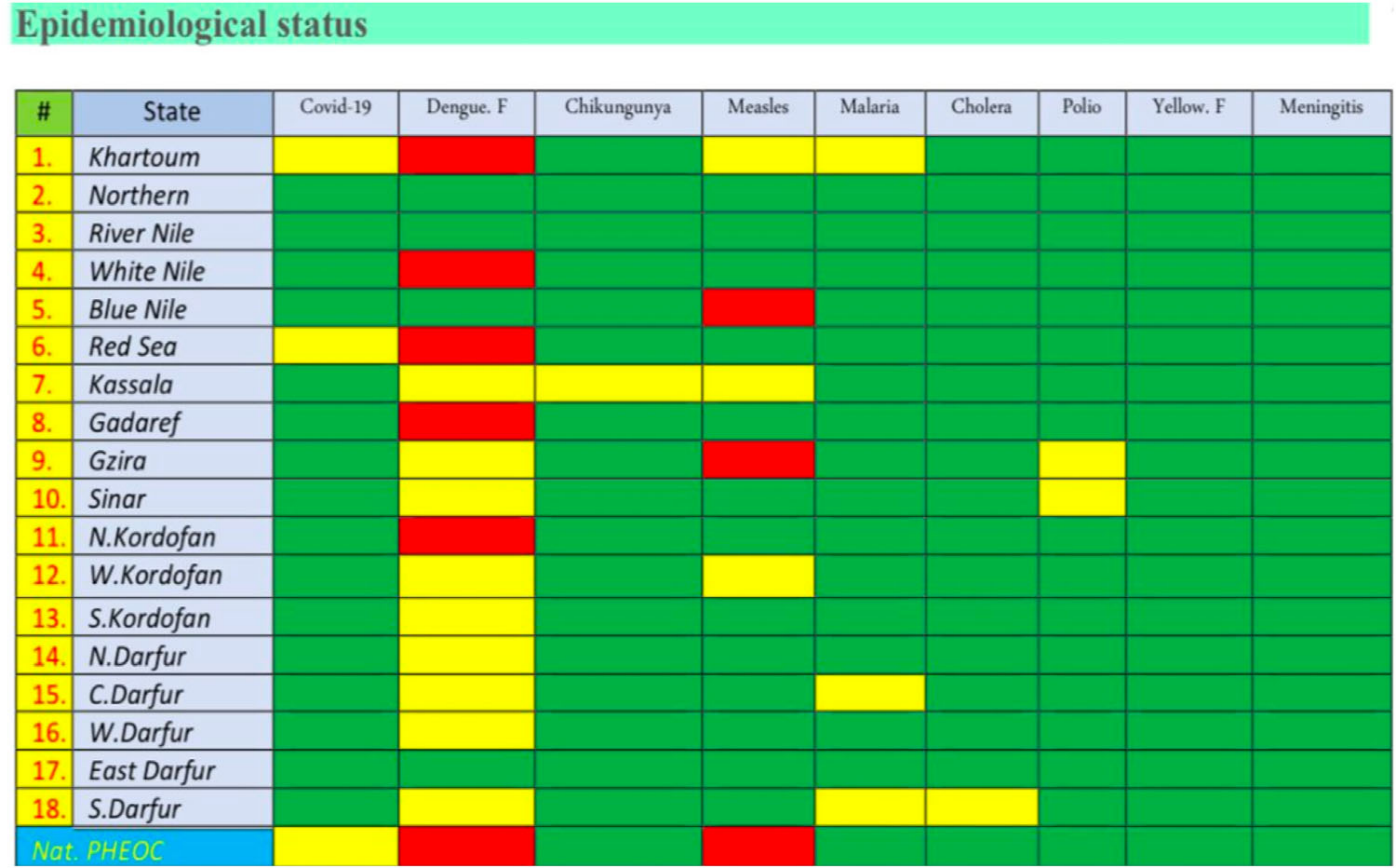
Sudanese Emergency Operating Center report, 4th Bulletin—published on July 4, 2023, green color indicate absence of outbreak, yellow indicates potential outbreak, and red active outbreak.

Prior to this conflict, Sudan has struggled with a high burden of communicable diseases (e.g., dengue cases reported from the capital Khartoum during late 2022–early 2023). This struggle is expected to remain given the impact that the conflict will have in the outbreak detection and response system. The Emergency Operation Center has already reported alarming rates of dengue fever in six states, and Measles in four states (Figure 3). The conflict is also expected to cause surges in the cases of malnutrition; the conflict has resulted in the destruction of the factory manufacturing malnutrition treatment meals and solutions [9]. Children will expectedly be at the most risk, but adult populations are equally expected to struggle if the war is to continue. The struggle of noncommunicable diseases’ patients is also expected to sustain by the reduced supply chain of chronic medications, reduced access to health facilities, and increased negative social determinants of health at every level.

The burden of mental health diseases and sexual and reproductive health (SRH) related issues often goes under‐reported in Sudan. The impact of the conflict on mental health might become significant as a result of the catastrophic events or damages in the assets, or torture and burglary acts practiced by RSF. The war has also brought spikes in gender‐based violence and numerous reported cases of rape and sexual assault by RSF soldiers, combined with the lack of access to abortion services, family planning, and HIV medications [10].

## HUMANITARIAN RESPONSE PRIORITIES

Although it is unlikely to happen anytime soon, peace is the urgently needed solution to preserving the health system in Sudan and protecting the lives and health of the Sudanese people. The national and international community must keep the following key considerations in responding to the ongoing conflict.

Restoration of the health system and continuity of service must ultimately remain the top priorities in response to the conflict. The response plans must prioritize certain health services, certain service delivery points, and certain affected populations. Emergency obstetric and newborn care, immunization, treatment of common childhood illnesses, infant and young child feeding, and malnutrition treatment, screening, top noncommunicable disease and other chronic diseases, injuries and accidents, mental health, and SRH must be prioritized in the current context of Sudan [11]. This is to be achieved via strategic readjustments of health facilities maps to support safer states that receive the most internally displaced people (such as Gezira state) while prioritizing the health priorities of the most vulnerable groups (including women and children).

Massive coordination is needed between the FMoH and its partners. The FMoH has to take the initiative in setting the health priorities, whereas “international partners” such as UN agencies should remain supportive. The recently amended Sudan Humanitarian Response Plan requires US$2.6 billion to be able to provide comprehensive life‐saving multisectoral assistance and protection services for 18.1 million people until the end of 2023 [8]. There also needs to be critical empowerment of civil society organizations (CSOs) and other local actors given the delays and restrictions aid agencies face in obtaining visas for their international staff, recognizing the vitality of establishing the required safety for nationals to operate is crucial. Experiences of other countries have used local actors for short‐term effectiveness, with successful examples from MSF in five countries [12]. This war presents an opportunity to build the long‐term capacity of locally based actors and CSOs. Lastly, health system response also must entail key political commitments to follow the International Humanitarian Law and to ensure the safety of operating staff and safe routes for emergency support medications and other supplies.

## CONCLUSION

An armed conflict has erupted in Sudan in April 2023 and has resulted in increased mortality and morbidity and massive population displacement. The impacts on Sudan's health system include closure of health facilities, suspension of services, reported shortages in supply, shrunken fiscal space and fiscal capacity, extension of the health leadership vacuum, disruption of health governance, increased risk for outbreaks, and spikes in malnutrition and GBV. Restoration of the health services and coordination among different actors remain the top recommendations, whereas conflict resolution is required to save lives and provide health services.

## AUTHOR CONTRIBUTIONS

*Writing of first draft; editing and review; proofreading*: Alhadi Khogali. *Writing of draft; editing; proofreading*: Anmar Homeida.

## CONFLICT OF INTEREST STATEMENT

We declare no conflicts of interest.

## FUNDING INFORMATION

The authors have received no funding for the development of this paper.

## ETHICS STATEMENT

This is a commentary. There is no need for ethical approval.

## Data Availability

Data sharing not applicable to this article as no datasets were generated or analyzed during the current study.
